# The influence of dopamine-beta-hydroxylase and catechol *O*-methyltransferase gene polymorphism on the efficacy of insulin detemir therapy in patients with type 2 diabetes mellitus

**DOI:** 10.1186/s13098-017-0295-0

**Published:** 2017-12-04

**Authors:** Tomislav Bozek, Antonela Blazekovic, Matea Nikolac Perkovic, Kristina Gotovac Jercic, Aleksandra Sustar, Lea Smircic-Duvnjak, Tiago F. Outeiro, Nela Pivac, Fran Borovecki

**Affiliations:** 1 Vuk Vrhovac University Clinic, Merkur University Hospital, Zagreb, Croatia; 2 Department for Functional Genomics, Center for Translational and Clinical Research, University of Zagreb School of Medicine, University Hospital Center Zagreb, Šalata 2, Zagreb, Croatia; 3 Division of Molecular Medicine, Rudjer Boskovic Institute, Zagreb, Croatia; 4 Department of Cardiology, University Hospital Center Rijeka, Rijeka, Croatia; 5 Department of Experimental Neurodegeneration, Center for Nanoscale Microscopy and Molecular Physiology of the Brain (CNMPB), Center for Biostructural Imaging of Neurodegeneration, University Medical Center Göttingen, Göttingen, Germany; 6 Max Planck Institute for Experimental Medicine, Göttingen, Germany

**Keywords:** Type 2 diabetes mellitus, Insulin detemir, COMT Val108/158Met polymorphism, DBH-1021C/T polymorphism, Hemoglobin A1c (HbA1c), BMI

## Abstract

**Background:**

Type II diabetes is an important health problem with a complex connection to obesity, leading to a broad range of cardiovascular complications. Insulin therapy often results in weight gain and does not always ensure adequate glycemic control. However, previous studies reported that insulin detemir is an efficient long-acting insulin with a weight sparing effect. The aim of this study was to determine the association of catechol *O*-methyltransferase (COMT) Val108/158Met and dopamine-beta-hydroxylase (DBH) 1021C/T polymorphisms with the effectiveness of insulin detemir in achieving glucose control and body weight control. Participants and methods: This 52-week observational study included 185 patients with inadequate glycemic control treated with premix insulin analogues, which were replaced with insulin aspart and insulin detemir, and 156 healthy controls. After DNA isolation from blood samples, genotyping of DBH-1021C/T polymorphism (rs1611115) and COMT Val108/158Met polymorphism (rs4680) was performed.

**Results:**

Our results confirmed that insulin detemir did not lead to weight gain. The most significant finding was that A carriers (the combined AG and AA genotype) of the COMT Val108/158Met achieved significantly better hemoglobin A1c (HbA1c) values compared to patients carrying GG genotype. No association between DBH-1021C/T genotypes and weight and/or glucose control was detected in diabetes patients or in healthy control subjects.

**Conclusions:**

This study showed that the presence of one or two A allele of the COMT Val108/158Met was associated with improved glycemic response, and with a better response to insulin detemir therapy in patients with type II diabetes, separating them as best candidates for detemir therapy.

**Electronic supplementary material:**

The online version of this article (10.1186/s13098-017-0295-0) contains supplementary material, which is available to authorized users.

## Background

Type II diabetes (T2DM) represents an important health problem, affecting a substantial percentage of the world population. It is designated by a chronic progressive course and a subsequent need for a long-term insulin therapy to achieve optimal glucose control. Achieving the recommended target values can prevent complications and improve outcomes of diabetes. Still, a substantial number of T2DM patients do not achieve optimal glucose control despite intensive insulin treatment [1]. International studies of T2DM patients have shown that many patients fail to achieve hemoglobin A1c (HbA1c) target values [2].

### Obesity and diabetes

The increase in the prevalence of T2DM in recent decades parallels the rise in obesity [3]. Obesity is associated with an increased risk of developing insulin resistance, which combined with inappropriate β-cells compensatory mechanisms may lead to T2DM [4]. However, the interplay between diabetes and obesity harbors a much more complex correlation. Insulin therapy often leads to weight gain and the concern is that 80–90% of T2DM patients are already obese before insulin treatment [5]. Weight gain increases the risk of coronary heart disease and cardiovascular complications in people with diabetes [6]. Glucose homeostasis and insulin production are greatly determined by obesity and body fat distribution, however the pathogenesis of obesity-induced insulin resistance has not been fully elucidated [7]. Growing evidence suggests that nutrients and hormonal signals converge and directly act on brain centers, leading to changes in energy metabolism and stable body weight over time [8]. There is evidence suggesting that these same signals act on the central nervous system (CNS) to regulate glucose metabolism independently [8].

### Dopamine-beta-hydroxylase (DBH)

Several neurotransmitters, such as dopamine, gamma-aminobutyric acid (GABA), serotonin and norepinephrine, as well as peptides and amino acids, are involved in the CNS’s regulation of energy and glucose homeostasis and in food intake regulation [9]. Dopamine is involved in weight regulation and food intake: it modulates different physiological functions including salt metabolism, which is associated with weight gain. Dopamine-beta-hydroxylase (DBH) is an enzyme localized within secretory vesicles of norepinephrine and epinephrine producing neurons and neurosecretory cells, where it catalyzes the conversion of dopamine to norepinephrine [10]. It has been shown that several *DBH* gene polymorphisms influence the DBH plasma activity [11] including DBH-1021C/T functional polymorphism that accounts for 35–52% of the inter-individual variations in plasma DBH activity [10–12]. Plasma DBH activity is under the genetic control of a single-nucleotide polymorphism (SNP), DBH-1021C/T, in the 5′ flanking region of the *DBH* gene. In this context T-allele is associated with reduced DBH plasma activity, in comparison to C-allele [10, 12]. For many years now, scientists are connecting the DBH enzyme activity with diabetes and other high risk phenotypes, but the underlying mechanisms are not yet fully elucidated [13, 14]. However, it is known that DBH knock down mice are presenting with an impaired glucagon response to hypoglycemia and elevated insulin levels [14].

### Catechol *O*-methyltransferase (COMT)

Catechol *O*-methyltransferase is one of the major enzymes involved in catecholamine and estrogen degradation [15]. Given the fact that both catecholamines and estrogen are associated with changes in metabolism and food intake, it is not surprising that changes of this enzyme may participate in the weight gain [16]. COMT is an important modulator in the catabolism of extraneural dopamine [17]. COMT removes toxic metabolites from the body, and regulates blood pressure via catecholamine metabolism. Changes in the COMT activity, associated with genetic variants in the *COMT* gene have consequences in various mechanisms connected with development of obesity, personality changes and behavior disturbances [18–22]. The most investigated polymorphism of the *COMT* gene, COMT Val108/158Met, consists of G-to-A transition on positions 158 or 108 of the *COMT* gene leading to the replacement of the amino acid valine (Val) with the amino acid methionine (Met) [23, 24]. This polymorphism is associated with a three- to fourfold variation in the COMT enzyme activity, with the Val (G) allele displaying higher and the Met (A) allele lower enzymatic activity [25, 26]. There is a well-established association between the COMT Val108/158Met polymorphism and abdominal obesity and blood pressure increase [27], human hypertension [28], and T2DM [29].

### Therapeutic challenges

The number of treatment options for T2DM has increased over the past two decades. As the understanding of the underlying pathophysiological mechanisms in T2DM increases, pharmacological possibilities have expanded to target novel physiologic mechanisms [30]. In spite of the above mentioned, many patients remain uncontrolled and the effectiveness of current therapies wanes over time [4]. There is also the problem of weight gain due to insulin therapy. Basal insulins are used to suppress uncontrolled hepatic glucose production and therefore have to be relatively long-acting. One of those long-acting insulins is detemir. Besides a low pharmacodynamic coefficient of variability [31], it exhibits anorexigenic features, probably through a complex interplay of its effects on the CNS [32] and on the finely tuned efferent and afferent signals between muscle, brain, liver, renal and adipose tissues [33].

### Aim

Compared to other basal insulins, patients treated with insulin detemir had reduced weight gain, which could be due to reduced energy intake rather than increased energy expenditure [34]. The aim of this study was to determine the association of COMT Val108/158Met and DBH-1021C/T polymorphisms with effectiveness of insulin detemir in achieving glucose control as well as body weight control.

## Methods

### Patients and control subjects

This 52-week observational monocentric study was conducted at the University Clinic for Diabetes, Endocrinology and Metabolic Diseases Vuk Vrhovac, Zagreb, Croatia. The study included 185 (70 male, 115 female) patients diagnosed with type 2 diabetes aged 20–80 years with inadequate glycemic control [HbA1c level from 7 to 11% (53–97 mmol/mol)] on a retrospective documented treatment with premix insulin analogues and 156 (52 male, 104 female) healthy control subjects, sampled during their routine laboratory check-ups. All subjects were Caucasians of Croatian origin. Patients treated with antipsychotic medications, those who had clinically significant gastroparesis, an end stage renal disease, severe chronic pancreatitis, a severe liver dysfunction with portal hypertension or cirrhosis, an inflammatory bowel disease (Crohn’s disease, ulcerative colitis), unregulated hypothyroidism or hyperthyroidism, a known malignant disease, who underwent bariatric surgery, or with a history of drug or alcohol abuse were not included in this study.

After selection and randomization of patients with type 2 diabetes, premixed insulin analogues were replaced with three doses of insulin aspart applied before main meals, and one dose of insulin detemir at bedtime, and followed for 52 weeks. Administration of metformin if not contraindicated was proceeded. A dose adjustment of insulin detemir and insulin aspart was performed according to glucose profile based on self-monitoring measurement and HbA1c level.

All recruited patients went through a comprehensive educational program, and became familiar with meal planning, exercising (compatible with their physical condition), glucose self-monitoring on regular basis four times per day, as well as with insulin dose adjustment according to American Diabetes Association (ADA) guidelines [35]. Preinclusion data was obtained retrospectively from medical records.

### Clinical measurements

All examinations were performed in the morning after an overnight fasting period by the same research nurses and physician-at the baseline visit on both, T2DM patients and healthy controls, and after 52 weeks only on T2DM patients. Body weight was measured using a balanced-beam scale and was expressed in kilograms (kg). Height was measured using a wall-mounted stadiometer and expressed in centimeters (cm). Body mass index (BMI) was calculated based on these measures as kilograms per square meter (kg/m^2^). Blood pressure was measured on the right arm after a resting period of 10 min in a sitting position with a mercury sphygmomanometer and expressed in millimeters of mercury (mmHg). Venous blood samples were collected for determination of biochemistry, lipid profile status and HbA1c, both on baseline and after a 52 week treatment period. Blood samples for DNA isolation and DBH and COMT gene polymorphism genotyping were taken at the end of the 52 week study period.

HbA1c was measured spectrophotometrically by turbidimetric immuno inhibition (Olympus AU600 Beckman Coulter, USA). Glucose, cholesterol and triglycerides in serum were measured by an enzymatic colorimetric method.

Written informed consent was obtained from all participants, after explaining the aims and procedures of the study, under guidelines approved by Ethics committee of the University of Zagreb School of Medicine and Clinical Hospital Merkur Zagreb. All studies have been carried out with the full cooperation of participants, adequate understanding, and have therefore been performed in accordance with the ethical standards of the Declaration of Helsinki.

### Molecular genetic analyses

DNA was isolated from whole blood using DNeasy Blood and Tissue Kit (Qiagen, Chatsworth, CA) according to manufacturer’s instructions. DNA extraction and genotyping were performed at Department for Functional Genomics, Center for Translational and Clinical Research, University of Zagreb School of Medicine, Croatia and at the Laboratory for Molecular Neuropsychiatry, Division of Molecular Medicine, Rudjer Boskovic Institute, Zagreb, Croatia. COMT Val108/158Met (rs4680) and DBH-1021C/T (rs1611115) polymorphisms were determined by ABI Prism 7300 Real time PCR System apparatus (Applied Biosystems, Foster city, California, USA), according to the procedures described by Applied Biosystems. The primers and probes were purchased from Applied Biosystems as TaqMan^®^ Drug Metabolism Genotyping Assay (C_25746809_50 for COMT) or TaqMan^®^ SNP Genotyping Assay (C_2535786_10 for DBH). All genotyping procedures were done blindly to clinical data. As a quality control for genotyping analyses, 5% of all samples were genotyped again.

### Statistical analysis

Baseline data was reported using descriptive statistics. The results, expressed as means (x) ± standard deviation (SD) or medians, were evaluated with Sigma Stat 3.5 (Jandell Scientific Corp. San Raphael, California, USA) using one-way and repeated measures analysis of variance (ANOVA) and t test, or with Kruskal–Wallis ANOVA on ranks, Mann–Whitney test, and Wilcoxon Signed Rank Test, when the normality of the data failed. The Hardy–Weinberg analysis was used to test the equilibrium of the population. The differences in the genotype frequencies were evaluated using the Chi square test. The level of significance was set to p value less than 0.05.

## Results

### Demographics

Demographic data for all 185 patients (70 male, 115 female) and 156 healthy controls (52 male, 104 female) are shown in Table 1. The mean age of studied population was 67.1 ± 8.01 years, with a mean duration of diabetes 16.2 ± 5.95 years. The mean age of healthy controls was 44.1 ± 11.6 years. Patients were treated with premix insulin analogues for a mean duration of 5.7 ± 2.8 years (time from initiation of insulin therapy to inclusion into this study), and were on an average daily dose of 0.72 Units/kg (Table 1).

**Table 1:** Demographic data for 185 patients with type 2 diabetes

Variable	Descriptive statistics				
	Mean	Median	Minimum	Maximum	Std. dev.
Age (years)	67.11	67.00	43.00	85.00	8.01
BW (kg) at baseline	84.13	83.00	54.00	128.00	13.94
BW after 52 weeks (kg)	83.78	83.00	55.00	120.00	13.32
BMI (kg/m^2^) at baseline	30.37	30.04	20.66	52.60	4.58
BMI after 52 weeks (kg/m^2^)	30.23	29.76	22.55	47.26	4.31
HBA1c (%) at baseline	8.58	8.50	6.20	12.80	1.02
HBA1c after 52 weeks (%)	7.78	7.70	5.10	11.90	1.11
Fasting glucose levels at baseline (mmol/L)	11.74	11.30	5.20	21.30	2.81
Fasting glucose levels after 52 weeks (mmol/L)	8.73	8.20	4.40	17.70	2.37
Detemir dose (units/day)	34.39	32.00	5.00	120.00	14.84
T2DM duration (years)	16.15	16.00	3.00	38.00	5.95
Premix dose (IU/day)	60.51	56.00	22.00	180.00	22.44
Detemir dose per kg (units/kg/day)	0.41	0.38	0.08	1.30	0.16

*BW* body weight, *BMI* body mass index

**Fig. 1:**
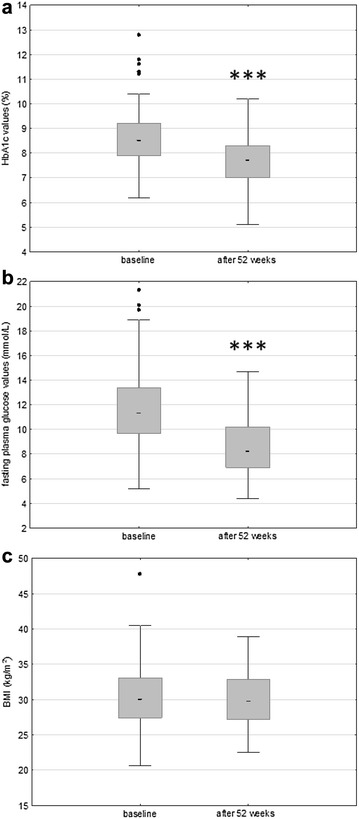
Changes in HbA1c, fasting plasma glucose values, and BMI due to 52-weeks of treatment. Central box represents the values from the lower to upper quartile and the middle line represents the median. The horizontal line extends from the minimum to the maximum value (non-outlier range) and the “far out” values (outliers) are displayed as separate points: **a** changes in HbA1c due to 52-week of treatment; **b** changes in fasting plasma glucose values due to 52-week of treatment; **c** changes in BMI due to 52-week of treatment. ***p < 0.001 vs. the baseline values

In patients with type 2 diabetes, a significant difference between HbA1c values [median 8.5 (min 6.2–max 12.8) vs. 7.7% (min 5.1–max 11.9) (69 vs. 61 mmol/mol); T = 1861; p < 0.001; Wilcoxon Signed Rank Test] and between fasting plasma glucose values [median 11.3 (min 5.2–max 21.3) vs. 8.2 (min 4.4–max 17.7) mmol/L; T = 972; p < 0.001; Wilcoxon Signed Rank Test] was observed, determined at baseline and at the end of the 52 week follow up period (Fig. 1). HbA1c values and fasting plasma glucose values were significantly decreased after 52 weeks of treatment compared to baseline values. At the end of the follow up period, 28.1% of patients on intensified insulin regimen with treatment of three times daily of insulin aspart and insulin detemir at bedtime, achieved HbA1c < 7.0% (< 53 mmol/mol). This reduction was obtained with a mean dose of 0.44 ± 0.19 Units/kg insulin aspart and 0.41 ± 0.16 Units/kg of insulin detemir, respectively. There was no significant decrease in BMI values in the whole patient list [median 30.04 (min 20.66–max 52.6) vs. 29.76 (min 22.55–max 47.26) kg/m^2^; T = 3944; p = 0,312; Wilcoxon Signed Rank Test] (Fig. 1c), or between female or male patients.

As expected, there was a significant difference in the BMI between patients [median 30.04 (min 20.66–max 52.6) kg/m^2^] and healthy controls [median 25.30 (min 15.6–max 54.00) kg/m^2^]; and in fasting plasma glucose values in T2DM patients [median 11.3 (min 5.2–max 21.3) mmol/l] vs. healthy controls [median 5.1 (min 4.00–max 6.90) mmol/l], (p < 0.001, Mann–Whitney test).

### Weight change due to insulin therapy

Based on the difference in weight change in response to therapy, patients were divided into three groups: groups with weight gain, weight reduction or no change in weight after the 52-week follow up period. After 52 weeks, a body weight reduction (3.4 ± 3.2 kg) was observed in 73 patients (39.5%); no change in body weight was found in 52 patients (28.1%); and 60 patients (32.4%) gained weight (3.3 ± 2.2 kg). The results revealed that the weight change and the starting BMI values were inversely proportional, since the group who lost weight had the highest starting BMI [mean 31.2 ± 5.2; median 30.35 (min 23.18–max 52.60) kg/m^2^], while the observed group who gained weight had the lowest starting BMI [mean 29.1 ± 4.2; median 29.21 (min 20.66–max 40.01) kg/m^2^]. The difference in BMI between these two groups was significant (U = 1725; p = 0.036; Mann–Whitney U test).

In order to explore the association of starting BMI with weight loss due to treatment, patients were additionally subdivided into four groups depending on the starting BMI (BMI < 27, 39 patients; BMI 27–29, 33 patients; BMI 29–31, 42 patients; BMI > 31, 71 patients). The change in HbA1c value was significant in all four groups of patients. The group with the lowest BMI (BMI < 27) needed significantly lower doses of insulin detemir per kg than other groups [median 0.32 (min 0.13–max 0.77) vs. median 0.4 (min 0.085–max 1.30) Units/kg] (U = 1215; p < 0.001 Mann–Whitney U test) to obtain adequate glycemic control.

### Association between COMT Val108/158Met and DBH-1021C/T polymorphisms and clinical parameters

No significant deviation from the Hardy–Weinberg equilibrium was found for either COMT Val108/158Met (χ^2^ = 1.602) or DBH-1021C/T (χ^2^ = 0.079) genotypes.

The frequency of the COMT Val108/158Met or DBH-1021C/T genotypes in T2DM patients and in healthy control subjects is presented in Table 2. The frequency of the COMT Val108/158Met (χ^2^ = 0.389; p = 0.824) or DBH-1021C/T (χ^2^ = 0.097; p = 0.952) genotypes did not differ significantly between healthy controls and patients with type 2 diabetes. A similar genotype distribution was found between female patients and female controls for COMT Val108/158Met (χ^2^ = 0.853; p = 0.653) and DBH-1021C/T (χ^2^ = 0.100; p = 0.951) genotypes, and between male patients and male controls for COMT Val108/158Met (χ^2^ = 0.147; p = 0.929) and DBH-1021C/T (χ^2^ = 1.041; p = 0.594) genotypes, respectively (Table 2).

**Table 2:** Distribution of the COMT Val108/158Met and DBH-1021C/T genotypes in patients with type 2 diabetes (T2DM patients) and healthy controls, and in subjects subdivided according to gender

	COMT Val108/158Met			DBH-1021C/T		
T2DM/controls	AA	AG	GG	CC	CT	TT
T2DM patients	39 (21.1%)	101 (54.6%)	45 (24.3%)	113 (61.1%)	64 (34.6%)	8 (4.3%)
Healthy controls	36 (23.1%)	80 (51.3%)	40 (25.6%)	94 (60.3%)	56(35.9%)	6 (3.8%)
χ^2^ test	χ^2^ = 0.389; df = 2; p = 0.824			χ^2^ = 0.097; df = 2; p = 0.952		
T2DM women	25 (21.7%)	61 (53.1%)	29(25.2%)	68 (59.1%)	42 (36.5%)	5 (4.4%)
Healthy women	27 (26%)	49 (47.1%)	28 (26.9%)	63 (60.6%)	36 (34.6%)	5 (4.8%)
χ^2^ test	χ^2^ = 0.853; df = 2; p = 0.653			χ^2^ = 0.100; df = 2; p = 0.951		
T2DM men	14 (20%)	40 (57.1%)	16 (22.9%)	45 (64.3%)	22 (31.4%)	3 (4.28%)
Healthy men	9 (17.3%)	31 (59.6%)	12 (23.1%)	31 (59.6%)	20 (38.5%)	1 (1.9%)
χ^2^ test	χ^2^ = 0.147; df = 2; p = 0.929			χ^2^ = 1.041; df = 2; p = 0.594		

There were no significant differences in the BMI values, body weight, fasting plasma glucose, or HbA1c in T2DM patients subdivided into carriers of the AA, GA and GG genotypes of the COMT Val108/158Met (Table 3), or when patients were subdivided into carriers of the CC, CT and TT of the DBH-1021C/T (Table 3), neither at baseline nor after 52 week treatment with insulin detemir. In healthy control subjects no significant differences were detected in BMI, body weight or fasting glucose values when subjects were subdivided into carriers of the COMT Val108/158Met AA, GA and GG genotypes or carriers of the DBH-1021C/T CC, CT and TT genotypes (Additional file 1: Table S1).

**Table 3:** Values of BMI, body weight, fasting glucose levels and HbA1c in in patients with type 2 diabetes (T2DM patients) subdivided into carriers of the COMT Val108/158Met and DBH-1021C/T genotypes

	At baseline			After 52-week treatment period		
COMT Val108/158Met genotype (number of patients)	AA (39)	AG (101)	GG (45)	AA (39)	AG (101)	GG (45)
BMI (kg/m^2^)	29.41 (22.59–37.8)	30.02 (20.66–47.80)	30.04 (20.98–52.6)	29.28 (23.84–37.78)	30.10 (22.55–45.17)	29.39 (22.92–47.26)
Kruskal–Wallis ANOVA on Ranks	H = 0.137; df = 2; p = 0.934			H = 0.067; df = 2; p = 0.967		
Body weight (kg)	85.00 (63.00–106.00)	82.00 (54.00–127.00)	84.00 (59.00–128.00)	84.00 (64.00–108.00)	83.00 (55.00–120.00)	82.00 (59.00–115.00)
Kruskal–Wallis ANOVA on ranks	H = 0.662; df = 2; p = 0.719			H = 0.167; df = 2; p = 0.920		
Fasting glucose levels (mmol/L)	12.30 (7.00–18.90)	11.00 (5.20–19.70)	11.70 (8.20–21.30)	8.40 (4.40–17.70)	8.10 (4.60–15.90)	8.10 (5.40–14.50)
Kruskal–Wallis ANOVA on ranks	H = 2.116; df = 2; p = 0.347			H = 0.397; df = 2; p = 0.820		
HbA1c	8.4 (7.00–11.20)	8.4 (6.20–11.80)	8.5 (7.20–12.80)	7.7 (6.20–10.20)	7.6 (5.10–10.5)	8.00 (5.80–11.90)
Kruskal–Wallis ANOVA on ranks	H = 2.684; df = 2; p = 0.261			H = 4.965; df = 2; p = 0.084		
	At baseline			After 52-week treatment period		
DBH-1021C/T genotype (number of patients)	CC (113)	CT (64)	TT (8)	CC (113)	CT (64)	TT (8)
BMI (kg/m^2^)	29.88 (20.66–47.80)	30.04 (22.19–52.60)	34.26 (26.47–35.51)	30.10 (22.55–45.17)	29.40 (22.60–47.26)	30.86 (27.14–34.26)
Kruskal–Wallis ANOVA on ranks	H = 1.426; df = 2; p = 0.490			H = 1.018; df = 2; p = 0.601		
Body weight (kg)	82.00 (58.00–127.00)	84.50 (54.00–128.00)	89.00 (72.00–110.00)	82.00 (57.00–120.00)	83.50 (55.00–115.00)	87.00 (72.00–106.00)
Kruskal–Wallis ANOVA on ranks	H = 1.279; df = 2; p = 0.527			H = 1.403; df = 2; p = 0.498		
Fasting glucose levels	11.10 (5.20–20.10)	11.5 (7.10–21.30)	11.9 (9.10–12.80)	8.25 (5.20–15.90)	8.25 (4.40–17.70)	7.10 (6.20–11,10)
Kruskal–Wallis ANOVA on ranks	H = 1.073; df = 2; p = 0.585			H = 0.373; df = 2; p = 0.830		
HbA1c	8.50 (6.50–12.80)	8.40 (6.20–11.80)	8.65 (7.80–10.20)	7.70 (5.90–11.90)	7.8 (5.10–10.30)	7.7 (6.20–8.60)
Kruskal–Wallis ANOVA on ranks	H = 0.612; df = 2; p = 0.736			H = 0.302; df = 2; p = 0.860		

* Data are presented as median and minimum and maximum

**Fig. 2:**
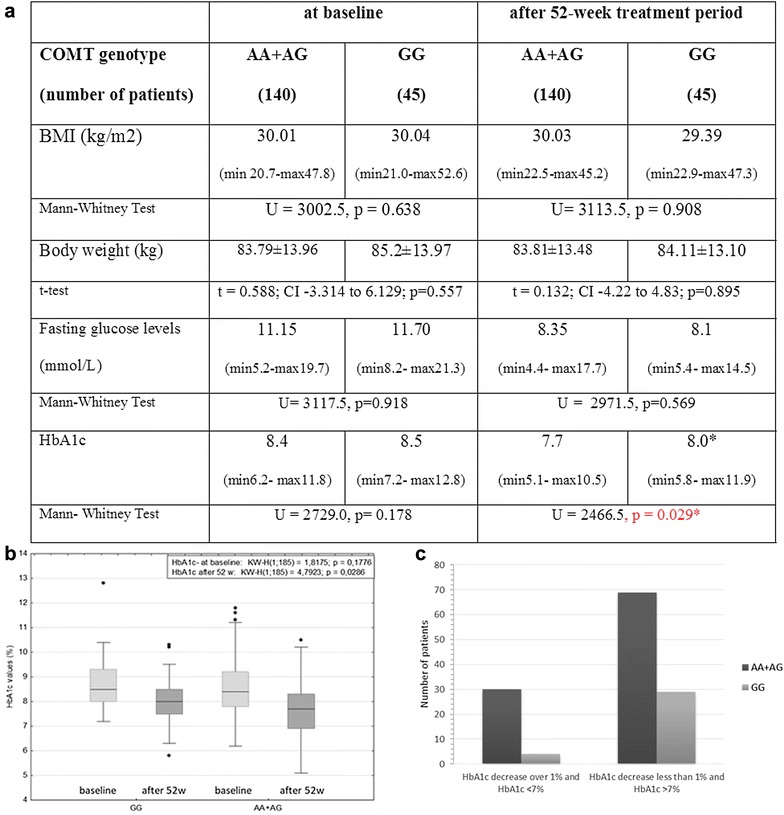
Association between COMT Val108/158Met A-carriers and clinical parameters: **a** Values of BMI, body weight, fasting glucose levels and HbA1c in T2DM patients subdivided into COMT Val108/158Met A-carriers and GG homozygous group; *data are presented as median and minimum and maximum. **b** The change in HbA1c in subjects subdivided into the A-carriers and GG homozygous genotype of the COMT Val108/158Met; central box represents the values from the lower to upper quartile and the middle line represents the median. The horizontal line extends from the minimum to the maximum value (non-outlier range) and the “far out” values (outliers) are displayed as separate points; **c** difference in the COMT Val108/158Met frequency in subjects subdivided according to the HbA1c decrease

Although there was no significant association between COMT Val108/158Met polymorphism and changes in BMI, body weight, fasting glucose levels and HbA1c, patients with T2DM were additionally subdivided into COMT Val108/158Met A carriers (i.e. subjects carrying the combined AG and AA genotypes) vs. GG homozygotes. COMT A carriers achieved significantly better HbA1c values after the 52 week treatment compared to patients carrying the GG genotype (A carriers: mean 7.55% (59 mmol/mol); median 7.70% (min 5.10–max 10.50) vs. GG genotype: mean 8.10% (65 mmol/mol) median 8.00% (min 5.80–max 11.90) (U = 2466.5; p = 0.029; Mann–Whitney test)) (Fig. 2a, b). This difference was not gender dependent. Among patients who had HbA1c-decrease over 1% and achieved HbA1c < 7% (< 53 mmol/mol), the GG genotype of the COMT was less frequently present when compared to the patients with higher levels of HbA1c (χ^2^ = 4.2879; p = 0.039; χ^2^ test) (Fig. 2c).

Although a visible trend of unfavorable clinical values in correlation with DBH TT genotype was observed, DBH-1021C/T genotypes were not significantly associated with any of the measured variables, possibly due to low frequency of this particular genotype (Additional file 1: Table S2).

## Discussion

This study revealed that A carriers (i.e. the combined AA and AG genotype) of the COMT Val108/158Met polymorphism achieved significantly better HbA1c values after 52 weeks of treatment, compared to patients with T2DM carrying GG genotype. Although we expected to detect the association of COMT Val108/158Met and/or DBH-1021C/T polymorphism with the effectiveness of insulin detemir in achieving glucose control and body weight control, our results did not confirm any other significant association with BMI, body weight or fasting glucose values in patients with T2DM.

In our study, COMT Val108/158Met or DBH-1021C/T polymorphisms were not associated with T2DM. These data do not agree with a significant association found between COMT Val108/158Met and T2DM [36]; however this association was detected in the Asian population, which could have additional confounding factors. In contrast to our results, in a large Caucasian population COMT Val108/158Met was associated with T2DM [29], while DBH-1021C/T was significantly associated with T2DM and other clinical phenotypes responsive to peripheral sympathetic tone in a tissue-specific manner [37], implying that present study lacked the statistical power or the needed sample size to detect these associations. However, this was not the main goal of the study, since we evaluated the possible association between COMT Val108/158Met and DBH-1021C/T polymorphisms and detemir-induced control of glucose control and body weight.

Subjects diagnosed with T2DM have a two- to fourfold higher chance of developing a serious cardiovascular outcome compared to those without diabetes [38]. Weight gain is one of the major problems associated with insulin therapy. The vast majority of patients with T2DM are resistant to insulin and have associated significant cardiovascular risk factors. Hyperglycemia is considered as a principal cause of diabetic complications. Elevated blood glucose levels in patients with diabetes increases the rate of glycation, a nonenzymatic process of reducing sugars with free amino groups of proteins, lipids and amino acids [39]. Glycated substances can be further modified in compounds called advanced glycation end products (AGE) which can trigger inflammatory reactions leading to atherosclerosis, kidney tissue damage, damage to small vessels in the eye and other major complications of diabetes acids [39]. Glycation is a process whose significance has recently been revealed also in many other diseases, including neurodegeneration [40]. Although at lowered blood glucose levels the sugars will be released from the amino groups, it is argued that most of the risk factors can be successfully controlled but the contribution of decreasing hyperglycemia is lower than expected [41, 42]. Increment of 1% in HbA1c increased the risk of cardiovascular disease mortality by 53% in type 1 diabetic patients, but only by 7.5% in patients with T2DM [41, 42]. Based on those data, it can hardly be expected that lowering of HbA1c of 1–2% alone is sufficient to significantly decrease the mortality risk in people with T2DM [41, 43]. On the other hand, weight loss represents one of the main goals of therapy in overweight patients with T2DM [44]. Clinical studies demonstrate that therapeutic benefit rises with increasing weight loss, but even losses as low as 0.45–4 kg have positive effects on metabolic control, cardiovascular risk factors and mortality rates [44]. In the present study, at the end of the 52 week of treatment, the main cardiovascular risk factors were significantly reduced for patients with T2DM. There was a significant decline in mean HbA1c value and in mean fasting plasma glucose value, which corresponds to the fundamental function of insulin. We did not observe weight gain which could be expected due to insulin therapy. These data agree with the previous known weight sparing effect of insulin detemir in comparison to other basal insulins [33, 45]. Zafar et al. [45] showed a dose-dependent weight gain of patients treated with insulin detemir. Since our patients had adjusted doses of detemir and aspart insulin, according to the glucose profile, it was not possible to detect a correlation between weight gain and insulin detemir dose. However, our results revealed that the group with the smallest BMI (BMI < 27) at baseline needed significantly lower doses of insulin detemir than other groups to obtain adequate glycemic control.

Some of the known cardiovascular risk factors, such as obesity and hypertension, are in part genetically determined, but the entire array of specific genes remains unidentified [46]. Our results showed different patterns of weight change and differences in achieving adequate glucose control in patients treated for 52 weeks with insulin detemir. Since COMT Val108/158Met and/or DBH-1021C/T polymorphisms are implicated in cardiovascular, sympathetic, and endocrine pathways [37, 47], we expected that treatment induced differences in weight change and glucose control might be associated with these polymorphisms. In line with other data that failed to show an association of COMT polymorphisms with weight, BMI, or obesity risk [48, 49], no significant association between COMT Val108/158Met or DBH-1021C/T genotypes and the change in body weight was detected. Still, our data showed that patients with TT genotype of the DBH-1021C/T or with AA genotype of the COMT Val108/158Met achieved a slight BMI decline, since there was a trend that did not reach the level of statistical significance. These results are partly consistent with previous reports showing an association between GG genotype with a fat-BMI [29], and with a slight decrease in percentage of body fat in AA carriers [49]. Although an association of COMT Val108/158Met genotypes with abdominal obesity and high blood pressure was found in Swedish men, connecting AA genotype with a higher risk of abdominal obesity, they failed to find a significant correlation to BMI [27]. These results only underline the ambiguous impact of COMT polymorphism on obesity.

On the other hand, our results showed that patients with best glycemic response were predominantly COMT Val108/158Met A carriers (i.e. carriers of the combined AA and AG genotypes). They achieved significantly better HbA1c values after the 52-week treatment compared to patients carrying the GG genotype, pointing to the fact that presence of one or two A allele of the COMT Val108/158Met could be associated with a better response to insulin detemir therapy. This finding differs from the data from a recent study that reported an association of the G allele with lower values of HbA1c [50], but is partly consistent with the results from a male obesity study in Denmark in which the GG genotype was associated with impaired glucose tolerance and high fat BMI [29, 50].

In our study, and in line with previous data [51], DBH-1021C/T polymorphism was not associated with changes in BMI values, body weight, fasting plasma glucose, or HbA1c in T2DM patients, or with BMI, body weight and fasting glucose values in healthy controls. A preclinical study showed that DBH deficient mice exhibit hyperinsulinemia, lower plasma glucose levels, and insulin resistance [52]. However, in our study there was no association between fasting glucose levels and DBH-1021C/T and/or COMT Val108/158Met polymorphisms.

Estrogen regulates COMT activity [53], and women have lower COMT enzymatic activity than men and genotype effect was more pronounced in males than in females [25]. In agreement with our previous results including 1058 healthy Caucasian subjects [54], a lack of gender dependent differences in the COMT Val108/158Met genotype frequency was detected in healthy controls, or in patients with T2DM (present study); and genotype effect was similar in both sexes. The differences with previous study [25], might be explained by the different diagnoses, different number and different ethnicities of subjects included.

In line with well-known effects of insulin detemir as a well-tolerated and effective long acting insulin [55], our data confirmed its highly beneficial weight sparing effect, especially in overweight patients, which is consistent with previous studies [34, 45], showing that treatment with insulin detemir was associated with less pronounced weight gain.

In conclusion, our results revealed that the presence of one or two A allele of the COMT Val108/158Met was associated with improved glycemic response, since the carriers of the combined AA and AG genotypes achieved significantly better HbA1c values after the 52-week insulin detemir treatment compared to patients carrying the GG genotype. These data suggest a protective effect of the COMT Val108/158Met A allele and a better response in A carriers to insulin detemir therapy. As far as we are aware, this is the first study to reveal a lack of significant association between DBH-1021C/T and effectiveness of insulin detemir in achieving glucose control as well as body weight control.

## Additional file

**Additional file 1.** Additional tables.

## Abbreviations

COMT: catechol *O*-methyltransferase
DBH: dopamine-beta-hydroxylase
HbA1c: hemoglobin A1c
T2DM: type II diabetes mellitus
GABA: gamma-aminobutyric acid
ADA: American Diabetes Association
BMI: body mass index
