# Supplementary material for: The influence of dopamine-beta-hydroxylase and catechol O-methyltransferase gene polymorphism on the efficacy of insulin detemir therapy in patients with type 2 diabetes mellitus
Source: Diabetol Metab Syndr. 2017 Dec 4;9:97. doi: 10.1186/s13098-017-0295-0 (PMC5716004; doi:10.1186/s13098-017-0295-0)
Supplement: Supplementary file 1 — Additional file 1. Additional tables. [file 13098_2017_295_MOESM1_ESM.docx]

Additional data:

Additional Table S1. BMI, BW and fasting glucose values in healthy controls subdivided according to the COMT Val108/158Met or DBH -1021C/T genotypes

| Healthy controls | COMT Val108/158Met | | | DBH -1021C/T | | |
| --- | --- | --- | --- | --- | --- | --- |
| (number of healthy controls) | AA  (36) | AG  (80) | GG  (40) | CC  (94) | CT  (56) | TT  (6) |
| BMI (kg/m^2^) | 25.80  (min18.5-max54.0) | 24.85  (min15.6-max39.0) | 25.80  (min18.2-max35.9) | 25.65  (min18.5-max54.0) | 24.35  (min15.6-max35.9) | 24.05  (min23.1-max34.1) |
| Kruskal Wallis ANOVA on Ranks | H=1.535; df=2; p=0.464 | | | H=2.904; df=2; p=0.234 | | |
| Body weight (kg) | 76.50  (min54.0-max215.0) | 78.25  (min40.0-max110.0) | 77.50  (min50-max107.0) | 79.75  (min50.0-max215.0) | 74.50  (min40.0-max107.0) | 67.50  (min58.0-max108.0) |
| Kruskal Wallis ANOVA on Ranks | H =0.0269; df=2; p=0.987 | | | H=2.192; df=2; p=0.334 | | |
| Fasting glucose levels | 5.10  (min4.0-max6.9) | 5.20  (min4.0-max6.7) | 5.10  (min4.3-max6.4) | 5.10  (min4.0-max6.9) | 5.20  (min4.4-max6.7) | 5.10  (min4.9-max6.4) |
| Kruskal Wallis ANOVA on Ranks | H=0.508; df=2; p=0.776 | | | H=4.925 ; df=2; p=0.085 | | |

* Data are presented as median and minimum and maximum

Additional Table S2. Values of BMI, body weight, fasting glucose levels and HbA1c in T2DM patients subdivided into C carriers (the combined CC and CT genotype) and TT carriers of the DBH -1021C/T

|  | at baseline | | after 52-week treatment period | |
| --- | --- | --- | --- | --- |
| DBH -1021C/T genotypes  (number of patients) | CT+CC  (177) | TT  (8) | CT+CC  (177) | TT  (8) |
| BMI (kg/m2) | 29.76  (min20.7-max52.6) | 33.12  (min26.5-max35.5) | 29.70  (min22.5-max47.3) | 32.42  (min27.4-max34.3) |
| Mann-Whitney Test | U = 529.0; p = 0.228 | | U = 58.0; p = 0.226 | |
| Body weight (kg) | 82.00  (min54.0-max128.0) | 87.50  (min72.0-max110.0) | 83.00  (min55.0-max120.0) | 86.50  (min72.0-max106.0) |
| Mann-Whitney Test | U = 898.5; p = 0.298 | | U = 898.0; p = 0.300 | |
| Fasting glucose levels (mmol/L) | 11.30  (min5.2-max21.3) | 11.50  (min9.1-max12.8) | 8.20  (min4.4-max17.7) | 8.45  (min6.2-max11.1) |
| Mann-Whitney Test | U = 663.5; p = 0,589 | | U = 671.0; p = 0.624 | |
| HbA1c | 8.50  (min6.2-max12.8) | 8.65  (min7.8-max10.2) | 7.70  (min5.1-max11.9) | 7.75  (min6.2-max8.6) |
| Mann-Whitney Test | U = 833.5; p = 0.548 | | U = 833.5; p = 0.548 | |

* Data are presented as median and minimum and maximum
